# Analysis of adherence and dropout factors from psychosocial treatment among people with serious mental illness

**DOI:** 10.1192/j.eurpsy.2025.827

**Published:** 2025-08-26

**Authors:** M. O. Pires, C. Osório, S. Ramos, R. Quelhas

**Affiliations:** 1 Unidade Local de Saúde da Guarda, Guarda; 2 Unidade Local de Saúde do Tâmega e Sousa, Penafiel; 3 Unidade Local de Saúde de Matosinhos, Matosinhos, Portugal

## Abstract

**Introduction:**

Psychosocial treatment is a crucial component of the rehabilitation process for individuals with serious mental illness (SMI). However, adherence to these interventions is often low, with high dropout rates posing challenges to successful outcomes. Understanding the factors that influence patient adherence, and dropout is essential for improving program efficacy and patient well-being.

**Objectives:**

This study aims to analyze the demographic, clinical, and motivational factors that contribute to adherence or dropout from the “*Programa Mais de Perto*” (PMDP), a psychosocial rehabilitation initiative for individuals with SMI, in order to enhance patient retention and optimize therapeutic outcomes.

**Methods:**

The study included all patients with SMI enrolled in the PMDP activities until December 2023. Data were collected through clinical file reviews and telephone interviews using a structured questionnaire, including the World Health Organization Disability Assessment Schedule (WHODAS 2.0). Demographic and clinical variables, as well as patient-reported experiences, were analyzed to identify factors associated with continued participation or dropout.

**Results:**

The study initially included 87 patients, of whom 41 adhered to the program and 46 dropped out. However, after further filtering and questionnaire analysis, a subset of 36 patients was evaluated in detail (22 in the adherence group and 14 in the dropout group). The adherence group had lower disability scores on WHODAS 2.0 and reported higher intrinsic motivation, such as improving physical and mental well-being. In contrast, the dropout group highlighted external pressures from mental health professionals as reasons for initial participation and cited fatigue, lack of integration, and distance from the program as reasons for discontinuation. *Tai Chi Chuan* was the most popular activity (39% participation), while the *Book Club* had the lowest engagement (3%).

**Conclusions:**

Intrinsic motivation is a key predictor of adherence to psychosocial rehabilitation programs, while external pressures may increase dropout risk. Enhancing flexibility, offering a wider range of activities, and fostering peer support systems could improve retention rates. These findings reinforce the importance of adapting psychosocial interventions to the needs and motivations of individuals with SMI for better long-term outcomes.

**Disclosure of Interest:**

None Declared

